# Attributes and circumstances that induce inappropriate health services demand: a study of the health sector in Brazil

**DOI:** 10.1186/s12913-015-0728-0

**Published:** 2015-02-18

**Authors:** Djalma S Guimarães, Eduardo JO Soares, Gileno Ferraz Júnior, Denise D Medeiros

**Affiliations:** Production Engineering Department, Federal University of Pernambuco, Recife, Brazil; Production Engineering Department, University of the State of Mato Grosso, Barra do Bugres, Brazil; Logistics Departament, University of Pernambuco, Nazaré da Mata, Brasil

**Keywords:** Health demand, Health system and logistic regression

## Abstract

**Background:**

The current economic and social context has required health systems to provide top quality services and to be efficient in controlling costs. An obstacle to achieve these goals is the inappropriate health services demand. This study aims to present these inappropriate health service demand determinants from data on telephone calls made to a medical advice call centre.

**Methods:**

This study used a Brazilian medical advice call centre data sample in the period of November and December 2012 (n = 19690), which supplied data on the user’s initial request, the physician’s recommendation, information on the patient and circumstances (the day and time of the day of the telephone call). The convergence between user intent and medical recommendation consists in adequate demand; otherwise the divergence consists in an inadequate one. In this way, using a logistic regression model, the critical factors that determine inappropriate health services request could be estimated.

**Results:**

In general, the user’s initial intent is the most critical for the inappropriate health system demand occurrence: the greater the complexity of the patient’s initial intent, the greater the chance the intent is wrong: (OR: 1.160; 95% CI: 1.113-1.210). With regard to the social characteristics, men are more likely to make inappropriate requests (OR: 1.102; 95% CI: 1038-1169); as well as youngsters are more likely to use the system incorrectly (OR = .993; 95% CI: .992 - .994). Regarding the circumstances (day and time of the call), requests in the final hours of the day and on days close to the weekend are more likely to be the inappropriate ones (OR: 1.082 for each six hour increase; 95% CI: 1.049-1.116) and weekday (OR: 1.017 for each day increase; 95% CI: 1.002-1.032).

**Conclusions:**

The critical profile for the inappropriate use occurrence consists of males and youngsters, who use the health service in the final hours of the day and at weekends, and mainly want to use more complex services. A practical implication of this research is to provide health systems managers, supporting information to the most critical users in order to assist them in making a decision when asking for health care.

## Background

The world health industry is facing a dilemma due to the expected services quality and range standards rise while at the same time it is under pressure to minimize costs due to the less favorable economic environment in several regions of the world.

One of the health system inefficiency causes is inappropriate requests for services since a significant portion of the health care systems expenses is set aside for procedures that are considered unnecessary. The patient’s initial intent on seeking a medical service may differ from the action that would be more recommended, a fact which is a burden on the system costs [1-7]. In United States for example, Bogdan et al. [2], using studies about medical advice call center, argue that changes from patients’ original healthcare intents had a potential net savings of $54.77 per patient, and Bunik et al. [4] pointed out that the estimated savings per call, based on local costs, was $42.61. One of the factors that causes callers’ error in judgment is that they lack appropriate information [8,9].

Given the information and communication technologies (ICT) development in recent decades, the health services inappropriate use problem has got new tools to minimize its harmful effects, the Mobile-health (m-health) tools [9-15], and medical advice call centers [2,4,16-20], are tools that give information to patients in order to assist them in making decision when asking for health services.

The potential request from the health system is not usually the appropriate request that would meet the patient’s real needs [1,5-7,21,22]. The medical advice online platform makes it possible for the user to obtain information that leads him/her to making a more appropriate use of available health services [2,19,23].

One indication of the initial requests inappropriateness and the caller’s real intent is presented by Bunik et al. [4], according to which two-thirds of cases in which the intent was to ask for a patient to go to an emergency department were identified as not necessary. Therefore, requests for more intensity of care in some cases lead to unnecessary actions.

Another aspect highlighted by the literature that influences inappropriate requests for health services is the individual’s age. Turner [24], Carret et al. [5], and McHale et al. [7] show that a higher percentage of inappropriate requests occurs with respect to youngsters.

Regarding gender, McHale et al. [7] found that males had a higher percentage of inappropriate use of emergency services. However, other previous studies point in the opposite direction [25-28], including a study conducted in Brazil [21].

With regard to the time factors of when requests for health services are made, some authors identified that phone calls requesting incorrect services were less intense in the early hours of the day [7,21,28,29]. The day of the week on which the user asks for health care is also considered when determining a caller’s misplaced intent. Pereira, et al. [26], Bianco et al. [29]; Afilalo et al. [30] and McHale et al. [7] emphasize that there is usually a higher percentage of callers seeking inappropriate use of emergency services at weekends and on public holidays.

According to Azevedo et al. [31] in Brazil there is an overvaluation of hospitals because primary care cannot meet on a regional basis, continued and systematized the most curative and preventive health system needs, so that patients are directed to the major hospitals in search of care for diverse needs. This feature contributes to the inappropriate use of health services and compromises the efficiency, quality and user satisfaction, according to research from the Brazilian Federal Council of Medicine [32] 93% of respondents considered the service of public health and supplemental in Brazil fair, poor or very poor. Carret et al. [5] states that 24.2% of the use of emergency in a medium-sized city in Brazil if they configured not needed, i.e., inadequate demand situation.

In this context, the aim of this study was to identify factors that determine inappropriate demand for health services in Brazil, based on data from telephone calls made to a medical advice call center. We also point out the impact on costs of health care for its appropriate use. Although inappropriate requests for healthcare services is a known problem in the world, in the literature there are few studies on this to be found. Thus, this study may provide important information for health managers.

## Methods

The data used in the research are information items contained in medical advice reports dealt with by phone which contain the following information: caller’s original plan for healthcare, the advisory call center medical recommendation, time of day and day of phone call, patient’s gender and age. It is important to consider that call center’s attendant is recommended to speak with parents or guardians if a young child or teenager (0-15 years old) make a call, in this sense, the decision to request a call center service lies with parents and guardians. These items of information were provided by the MedAlliance Company, a medical advice services provider, via call center switchboards, located in Recife, Brazil. This company provides medical counseling services to numerous companies of health insurance across Brazil and the healthcare workers that provide the medical advice are physicians themselves. For example, a user that is not feeling well can call the counseling center and be attended by a doctor in his/her immediate need. Patients may or may not follow the recommendations.

This information comprises all the calls received by the medical advice switchboard in the months of November and December 2012, a total of 19,690 observations. The service platform from supplier data follows the aloof medical care services structure presented by Akter et al. [19].

Variables extracted from the data set were: the patient’s initial intent for healthcare (1 = home care, 2 = Visit to a clinic, 3 = Hospital visit), the medical advice (1 = home care, 2 = Visit to a clinic, 3 = Hospital visit), gender (1 = Female, 2 = Male), age (continuous), the time of the phone call (00:01-06:00, 06:01-12:00, 12:01-18:00 p.m. and 18:01-24:00), and day (Monday, Tuesday, Wednesday, Thursday, Friday, Saturday, Sunday). The observations in which the service user refused to answer the question on what his/her original intent was when he/she phoned the call center (n = 108) were excluded from the sample.

The user’s original intention and the medical advice call center recommendations were divided into three categories as per Bogdan et al. [2]: home care (low intensity of care, including actions such as taking medication at home or resting); visit to a clinic (intermediate intensity, not including emergency actions, such as visiting a general practitioner, scheduling an appointment); and hospital visit (high intensity of care, including emergency actions and going to hospitals).

The user’s request was understood as follows: appropriate, when convergence between the original intent and recommendation occurs, and inappropriate, when the initial intent of the user’s request differs from the recommendation provided by the call center. Moreover, inappropriate request was classified as overestimated and underestimated request. In this sense, if the patient was initially intending to request a specific intensity of care but the medical adviser recommends a higher intensity of care it is classified as an underestimated request. Conversely, it is classified as an overestimated request.

The data were analyzed with SPSS software. Initial analysis was performed using chi-squared on the two groups of appropriateness. After that, it was used a binary logistic regression model, in which the request for the service is the dependent variable and the other requests are explanatory variables. In the regression model, the Stepwise Forward method was used. At each iteration, this inserts the significant variables of the model. In the end, the only parameters that remain are those that have a consistent relationship with the dependent variable.

To analyze the cost savings provided by the central medical advice, the difference between the cost of the patient original intention and the cost of medical recommendation was calculated (home care, visit to clinic and hospital visit). The values used to calculate were the current values in the Brazilian health service in December 2012.

The study was submitted to the Committee of Ethics in Research of the University of the State of Mato Grosso and is in compliance with the Helsinki Declaration. The information used in the research were obtained from a secondary dataset provided by the MedAlliance Company which having a care protocol that originates the data without user identification (patients). The rapport of the Committee of Ethics is contained in Letter No. 008/2014- REC/UNEMAT.

## Results

Table 1 presents some demographic information and sample characteristics of 19,582 phone calls dealt with by a medical advice system, as well as, presents the distribution of appropriate and inappropriate request according to these explanatory characteristics. Overall, 54.1% (n = 10,586) of the calls were considered as inappropriate requests and 45.9% (n = 8,996) as appropriate requests.

**Table 1:** **Sample profile and distribution of appropriate and inappropriate request according to explanatory variables**

**Characteristic**		**Total (n = 19582)**	**Appropriate (n = 8996)**	**Inapropriate (n = 10586)**	***P***
		**n (%)**	**n (%)**	**n (%)**	
Gender					
χ^2^ = 1.76; 1df	Female	12490 (63.8)	5693 (63.3)	6797 (64.2)	
	Male	7092 (36.2)	3303 (36.7)	3789 (35.8)	0.185
Age group (years)*					
χ^2^ = 129.56; 5df	0 – 15	5777 (29.5)	2358 (26.2)	3419 (32.3)	
	16 – 24	2181 (11.1)	999 (11.1)	1182 (11.2)	
	25 – 34	3836 (19.6)	1814 (20.2)	2022 (19.1)	
	35 – 44	2182 (11.1)	1003 (11.1)	1179 (11.1)	
	45 – 59	2581 (13.2)	1212 (13.5)	1369 (12.9)	
	60 plus	3025 (15.5)	1610 (17.9)	1415 (13.4)	<0.001
Time of the call					
χ^2^ = 52.64; 3df	00:01-06:00	1026 (5.2)	463 (5.1)	563 (5.3)	
	06:01-12:00	6233 (31.8)	3065 (34.0)	3168 (29.9)	
	12:01-18:00	6276 (32.1)	2888 (32.1)	3388 (32.0)	
	18:01-00:00	6047 (30.9)	2580 (28.7)	3467 (32.8)	<0.001
Initial intention (original plan)					
χ^2^ = 230.87; 2df	Home care	2019 (10.3)	864 (9.6)	1155 (10.9)	
	Visit to a clinic	5870 (30.0)	3182 (35.4)	2688 (25.4)	
	Hospital visit	11693 (59.7)	4950 (55.0)	6743 (63.7)	<0.001
Weekday					
χ^2^ = 5.12; 6df	Monday	2113 (10.8)	999 (11.1)	1114 (10.56)	
	Tuesday	3572 (18.2)	1675 (18.6)	1897 (17.9)	
	Wednesday	3218 (16.4)	1486 (16.5)	1732 (16.4)	
	Thursday	2727 (13.9)	1244 (13.8)	1483 (14.0)	
	Friday	2878 (14.7)	1301 (14.5)	1577 (14.9)	
	Saturday	2808 (14.3)	1276 (14.2)	1532 (14.5)	
	Sunday	2266 (11.6)	1015 (11.3)	1251 (11.8)	0.528

*Children and teenagers’ (0-15 years old) health service intended use is conducted by parents or guardians.

Medical advice call center users are predominantly women, the most common age range is from zero to fifteen years old and the most frequent time of day to use the service is between 06:00 am and 6:00 pm. The service user predominant intention is to seek an urgent/emergency service (59.7%). The inappropriate request was associated with the patient’s age (χ^2^ = 129.56; 5 df; P < 0.001), time of the call (χ^2^ = 52.64; 3 df; P < 0.001) and caller’s initial intention (χ^2^ = 230.87; 2 df; P < 0.001). Regarding age group we can see that both appropriate and inappropriate requests were more frequent in young children (<15 years old; 26% and 32.3% respectively). The proportion of patients whose inappropriate requests were more frequent was in the afternoon and evening calls (12:01 to 00:00) with 32.0 and 32.8% respectively. Furthermore, caller’s initial intent was more frequent with a higher intensity care (hospital visit 63.7%).

In the binary logistic regression analysis, we sought to examine the likelihood of inappropriate requests for healthcare service based on related socio-demographic characteristics, conditions of service (day and time of the call) and the initial intention complexity. The result can be seen in Table 2.

**Table 2:** **Regression results**

		**B**	**S.E.**	***P***	**OR**	**95,0% C.I. for EXP(B)**	
						**Lower**	**Upper**
Step 5(e)	Day	.017	.008	.027	1.017	1.002	1.032
	Time of Day	.079	.016	.000	1.082	1.049	1.116
	Gender	.097	.030	.001	1.102	1.038	1.169
	Initial Demand	.149	.021	.000	1.160	1.113	1.210
	Age	-.007	.001	.000	.993	.992	.994
	Constant	-.174	.068	.010	.840		

From the result of the regression model showed in Table 2, we can obtain the relationship of the dependent variables with the user’s correct request probability. The intention of the more intensive initial request increases by 16% the odds rate of inappropriate requests for the service (OR: 1.160; 95% CI: 1.113-1.210). The males are 10.2% more likely to make an inappropriate request (OR: 1.102; 95% CI: 1.038-1.169); the unit increment in the period of the day (6-hours) considered increased the odds rate of inappropriate demand by 8.2% (OR: 1.082; 95% CI: 1.049-1.116); and for the days of the week, each day represents an increase of 1.7% in the probability of inappropriacy (OR: 1.017; 95% CI: 1.002-1.032).

On the other hand, the age variable is a factor in reducing the probability of inappropriate requests, the increase of a year of life of the patient, decreases the likelihood of inappropriate intent demand by 0.7% (OR: 0.993; 95% CI: 0.992-0.994). It should be noted that in cases of care for individuals at an early age the request for healthcare is made by parents or guardians.

The proposed model is seen to be statistically significant because it rejects the null hypothesis (β0, β1, β2 and β3 = 0) in the likelihood ratio test. Therefore, the estimated parameters are significant. A fact endorsed by the low p - value (*P* <0.05).

Moreover, regarding the inappropriate demand, it is important to consider that callers can be in both directions, towards an overestimated or an underestimated need. In this sense, Table 3 presents the distribution of underestimated and overestimated inappropriate request according to these explanatory characteristics.

**Table 3:** **Distribution of underestimated and overestimated request according to explanatory variables**

***Characteristic***		***Underestimated (n = 2166)***	***Overestimated (n = 8420)***	***P***
		***n (%)***	***n (%)***	
Gender				
χ^2^ = 0.22; 1df	Female	1400 (64.64)	5397(64.10)	
	Male	766(35.36)	3023(35.90)	0.330
Age group (years)*	0-15	433(19.99)	2986(35.36)	
χ^2^ = 195.91; 5df	16-24	259(11.96)	923(10.96)	
	25-34	474(21.88)	1548(18.38)	
	35-44	294(13.57)	885(10.51)	
	45-59	344(15.88)	1025(12.17)	
	60 plus	362(16.71)	1053(12.51)	<0.001
Time of the call				
χ^2^ = 79.,03; 3df	00:01-06:00	86(3.97)	477(5.67)	
	06:01-12:00	781(36.06)	2387(38.35)	
	12:01-18:00	727(33.56)	2661(31.60)	
	18:01-00:00	572(26.41)	2895(34.38)	<0.001
Initial intention (original plan)	Home care	1155(53.32)	-	
χ^2^ = 6,710.32; 2df	Visit to a clinic	1011(46.68)	1677(19.92)	
	Hospital visit	-	6743(80.08)	<0.001
Weekday				
χ^2^ = 4.81; 6df	Monday	378(17.45)	1519(18.04)	
	Tuesday	367(16.94)	1365(16.21)	
	Wednesday	292(13.48)	1191(14.14)	
	Thursday	349(16.11)	1228(14.58)	
	Friday	306(14.13)	1226(15.56)	
	Saturday	255(11.77)	996(11.83)	
	Sunday	219(10.11)	895(10.63)	0.560

*Children and teenagers’ (0-15 years old) health service intended use is conducted by parents or guardians.

In general, 20.46% (n = 2,166) of the calls were considered as underestimated inappropriate requests and 79.54% (n = 8,420) as overestimated requests. The underestimated and overestimated inappropriate requests were associated with the patient’s age (χ^2^ = 195.91; 5 df, P < 0.001), time of the call (χ^2^ = 79.03; 3 df; P < 0.001) and caller’s initial intention (6,710.32; 2 df; P < 0.001). Through Table 3, we can see that overestimated requests were more frequent in younger children (0 to 15 years old) compared with underestimated request cases, and the latter were more frequent in younger adults (24 to 35 years old). Regarding time of the call, both overestimated and underestimated requests were more frequent in the interval from 06:01 am to 12:00 am. The proportion of overestimated was higher in hospital visit initial intent and lower in individuals who intended to visit a clinic (80.08 and 19.92% respectively). Furthermore, the frequency of underestimated was higher in home care initial intent and lower in individuals who intended to visit a clinic (53.32 and 46.68% respectively). Gender and weekday were not significantly associated with categories and the underestimated and overestimated demand.

Regarding impact on costs, we can indicate that, for Brazil in particular, given a cost of approximately US$ 76.50 per entry in an emergency unit and US$ 56.50 per medical consultation (including additional medical examinations), the cost to the health system on considering the intent of the initial request of all users analyzed in this study (n = 19.582) is US$1.226.169,50, while the cost to the system based on medical recommendations is US$ 890.779,00, possible savings of approximately 27.35%.

## Discussion

Reducing the number of inappropriate requests has great potential for savings in the health system [2,4,9,33]. The benefits, however, are not restricted to the cost aspect. Brazil since mid-2013 is experiencing a period of grass-roots dissatisfaction with some aspects of everyday life in the country, and the health system is one of the critical points of this dissatisfaction, and generated protests in the streets which were headline news worldwide. Much of the dissatisfaction is due to excessive waiting in line, the delay in being attended to and the conditions under which patients are seen in the healthcare system. Nor should it be lost from sight that minimizing inappropriate requests reduces waiting in line, Moreover, we can consider as benefits of the efforts of minimizing inappropriate requests, the possibility of reducing demands on the patient’s time which is currently taken up unnecessarily.

In this study it is evident that users who make an initial request for more intensity of care are a significant source of inappropriate requests, which corroborates the results of Bunik et al. [4]. Thus the development of a communication channel with the health service users through m-health systems and call centers, when used in an informed way, can assist them to make decisions about the healthcare service they need, reducing the number of inappropriate calls and reducing costs in the health system.

Regarding gender as a critical factor of inappropriate requests, there is no consensus in the literature. Some authors claim females are the critical factor for inappropriate requests [21,25-28]. However, the results of this study confirm the findings of McHale et al. [7] that men are more likely to generate inappropriate demand for health services. Thus, some campaigns have already been carried out by the Brazilian federal government to encourage male participation in preventive health practices [34].

The increase in patient age decreases the likelihood of inadequate demand 0.7% each year. The younger the patient, the greater the likelihood of inappropriate requests, thus confirming findings of other authors [5,7,21,24,28,32,35]. This user’s group of health service should receive special attention from health service providers in order to encourage their parents or guardians to use the tools of m-health and call centers with greater frequency. Furthermore, some device has been developed to provide information for children, Tate et al. [12] suggests the use of electronic media of the m-health type, such as SMS and applications (“apps”) and guidance on offering information and educative examples to school-age children, using preventive actions for obesity, for example, and highlights the main advantages of using such a platform as well as its challenges.

Regarding time, the end of the day, increases the probability of inappropriate requests by 8.2% in each period, which corroborates the results of McHale et al. [7] and Carret et al. [23]. In addition, there is a rise in the probability of inappropriate demands in the final days of the week which confirms the propositions of other authors [7,26,29,30].

Thus, an alternative to reduce unnecessary healthcare services is the existence of a system of preparedness in serving counseling and m-health tools to guarantee access to information anytime, as well as develop a trust relationship with the user so that the quality and prompt service are guaranteed.

Careful observation of inadequate demand occurrences for health services on one hand allows the observation of a direct relationship between inappropriate use and increased costs [2,4]. On the other hand, in cases where the demand is underestimated, the patient’s health is at risk, because in 20.46% of calls for central counseling the patient wished to sue insufficient services for his/her real need, which could significantly compromise his/her health and could generate higher future costs to the health system. In this way, the breakdown of inadequate demand profiles allows managers of health services personalize attention on possible risk groups.

According to Gentile et al. [22] one of the main reasons that leads a patient to seek an emergency department is the difficulty in obtaining an appointment with a general practitioner or receiving some medical advice. According to these authors, in France, the authorities solved to the problem by creating primary care units (PCUs) near hospitals, thus providing redirection to less urgent cases so as to avoid overcrowding emergency units. McHale et al. [7] also point out similar actions of the English authorities. In this context, the use of central medical advice by phone could promote redirect actions without the need for patients to go to emergency departments.

Thus, recognition of users more likely to misuse the health system can provide the opportunity to target education and information campaigns and incentives on how to use systems/platforms that provide further information to the health system user [9-11,36], so that in the act of taking a decision on what healthcare service to ask for, they take the decision that most closely matches their need, thereby reducing the inappropriate use of the system.

This research illustrates the potential for reducing costs in the health system, based on the identification of inappropriate use. Correct care of the patient flow can mitigate costs and minimize access problems. Thus, it points to the necessity of creating new access strategies to collaborate with the assistance flow optimization. The information in this profile can help health service managers in the targeting strategies of information and monitoring of users in order to minimize costs brought by such a group. In societies in which the demand for better health services are recurring, mapping the inadequate demand of critical users and the use of tools such as central medical counseling are basic tools for improving the system efficacy.

In this context, the challenge faced by health managers is to ensure that the user will adhere to the advisor recommendation or will use m-health systems. However, studies indicate that between 50% and 90% of patients reported having followed the advisory recommendations of medical advice call centers [2,16] and other studies present factors that influence the behavior of middle-aged users to accept using m-health system platforms [15].

## Conclusions

The main contribution of this study is to present a critical profile for the occurrence of inappropriate demands on the health service in Brazil. This information provides health systems managers with information which guides business strategies and aims at minimizing inappropriate requests in order to reduce costs and improve the service level of the system by reducing queues and unnecessary procedures. The medical advice call centers and other m-health tools can be vital in this effort since they aggregate information for the most critical users in order to assist them in making the most appropriate decision with regard to looking after their health.

## Abbreviations

ICT: Information and communication technologies
OR: Odds ratio
CI: Confidence interval

## References

[1] Martin A, Martin C, Martin PB, Martin PA, Green G, Eldridge S (2002). ‘Inappropriate’ attendance at an accident and emergency department by adults registered in local general practices: how is it related to their use of primary care?. J Health Serv Res Policy.

[2] Bogdan GM, Green JL, Swanson D, Gabow P, Dart RC (2004). Evaluating patient compliance with nurse advice line recommendations and the impact on healthcare costs. Am J Manag Care.

[3] Bezzina AJ, Smith PB, Cromwell D, Eagar K (2005). Primary care patients in the emergency department: Who are they? A review of the definition of the ‘primary care patient’ in the emergency department. Emerg Med Australas.

[4] Bunik M, Glazner JE, Chandramouli V, Emsermann CB, Hegarty T, Kempe A (2007). Pediatric telephone call centers: How do they affect health care use and costs?. Pediatrics.

[5] Carret ML, Fassa AC, Domingues MR (2009). Inappropriate use of emergency services: a systematic review of prevalence and associated factors. Cad Saude Publica.

[6] Durand A-C, Palazzolo S, Tanti-Hardouin N, Gerbeaux P, Sambuc R, Gentile S (2012). Nonurgent patients in emergency departments: rational or irresponsible consumers? Perceptions of professionals and patients. BMC Research Notes.

[7] McHale P, Madeira S, Hughes K, Bellis M, Demnitz U, Wyke S (2013). Who uses emergency departments inappropriately and when - a national cross-sectional study using a monitoring data system. BMC Med.

[8] Campbell RJ (2000). Consumer health, patient education, and the internet. The Internet Journal of Health.

[9] Mukherjee A, McGinnis J (2007). E-healthcare: an analysis of key themes in research. Int J Pharm Healthc Mark.

[10] Lund S, Hemed M, Nielsen BB, Said A, Said K, Makungu MH (2012). Mobile phones as a health communication tool to improve skilled attendance at delivery in Zanzibar: a cluster-randomised controlled trial. BJOG.

[11] Fischer HH, Moore SL, Ginosar D, Davidson AJ, Rice-Peterson CM, Durfee MJ (2012). Care by cell phone: text messaging for chronic disease management. Am J Manag Care.

[12] Tate EB, Spruijt-Metz D, O’Reilly G, Jordan-Marsh M, Gotsis M, Pentz MA (2013). mHealth approaches to child obesity prevention: successes, unique challenges, and next directions. TBM.

[13] Weinstein RS, Lopez AM, Joseph BA, Erps KA, Holcomb M, Barker GP (2013). Telemedicine, Telehealth, and mobile health applications that work: opportunities and barriers. Am J Med.

[14] Lee SSS, Xin X, Lee WP, Sim EJ, Tan B, Bien MPG (2013). The feasibility of using SMS as a health survey tool: an exploratory study in patients with rheumatoid arthritis. Int J Med Inform.

[15] Deng D, Mo X, Liu S (2014). Comparison of the middle-aged and older users’ adoption ofmobile health services in China. Int J Med Inform.

[16] O’Connell JM, Towles W, Yin M, Malakar CL (2002). Patient decision making: use of and adherence to telephone-based nurse triage recommendations. Med Decis Making.

[17] Kempe A, Bunik B, Ellis J, Magid D, Hegarty T, Dickinson LM (2006). How safe is triage by an after-hours telephone call center?. Pediatrics.

[18] Dunt D, Wilson R, Day SE, Kelaher M, Gurrin L (2007). Impact of telephone triage on emergency after hours GP Medicare usage: a time-series analysis. Aust N Z Health Policy.

[19] Akter S, D’ambra J, Ray P (2010). Service quality of mHealth platforms: development and validation of a hierarchical model using PLS. Electron Markets.

[20] Huibers L, Keizer E, Giesen P, Grol R, Wensing M (2012). Nurse telephone triage: good quality associated with appropriate decisions. Fam Pract.

[21] Carret M, Fassa A, Kawachi I (2007). Demand for emergency health service: factors associated with inappropriate use. BMC Health Serv Res.

[22] Gentile S, Vignally P, Durand A-C, Gainotti S, Sambuc R, Gerbeaux P (2010). Nonurgent patients in the emergency department? A French formula to prevent misuse. BMC Health Serv Res.

[23] Ivatury G, Moore J, Bloch A (2009). A doctor in your pocket: health hotlines in developing countries. Innovations: Technology, Governance, Globalization.

[24] Turner VF, Bentley PJ, Hodgson SA, Collard PJ, Drimatis R, Rabune C (2002). Telephone triage in Western Australia. Med J Aust – eMJA.

[25] Liu T, Sayre MR, Carleton SC (1999). Emergency medical care: types, trends, and factors related to nonurgent visits. Acad Emerg Med.

[26] Pereira S, Oliveira-e-Silva A, Quintas M, Almeida J, Marujo C, Pizarro M (2001). Appropriateness of emergency department visits in a Portuguese university hospital. Ann Emerg Med.

[27] Sarver JH, Cydulka RK, Baker DW (2002). Usual source of care and nonurgent emergency department use. Acad Emerg Med.

[28] Oktay C, Cete Y, Eray O, Pekdemir M, Gunerli A (2003). Appropriateness of emergency department visits in a Turkish university hospital. Croat Med J.

[29] Bianco A, Pileggi C, Angelillo IF (2003). Non-urgent visits to a hospital emergency department in Italy. Public Health.

[30] Afilalo J, Marinovich A, Afilalo M, Colacone A, Leger R, Unger B (2004). Nonurgent emergency department patient characteristics and barriers to primary care. Acad Emerg Med.

[31] Azevedo ALCS, Pereira AP, Lemos C, Coelho MF, Chaves LDP (2010). Organization of hospital emergency services: integrative research review. Rev Eletr Enf.

[32] Brazilian Federal Council of Medicine. [http://portal.cfm.org.br/images/PDF/apresentao-integra-datafolha203.pdf]

[33] Kile J, Healey BJ, Mcgowan M (2008). Charging for medical telephone care. Acad Health Care Manag J.

[34] Brasil, Ministério da saúde. Warning National Policy Integral Health Man, Brasília 2008. http://dtr2001.saude.gov.br/sas/PORTARIAS/Port2008/PT-09-CONS.pdf

[35] Sempere-Selva T, Peiró S, Sendra-Pina P, Martinez- Espin C, Lopez-Aguilera I (2001). Inappropriate use of an accident and emergency department: magnitude, associated factors, and reasons: an approach with explicit criteria. Ann Emerg Med.

[36] Bert F, Gualano MR, Brusaferro S, De Vito E, de Waure C, La Torre G (2013). Pregnancy e-health: a multicenter Italian cross-sectional study on internet use and decision-making among pregnant women. J Epidemiol Community Health.

